# 2,4-Di­chloro-1-iodo-6-nitro­benzene

**DOI:** 10.1107/S1600536814008733

**Published:** 2014-04-26

**Authors:** Xueshu Li, Sean Parkin, Hans-Joachim Lehmler

**Affiliations:** a The University of Iowa, Department of Occupational and Environmental Health, UI Research Park, Iowa City, IA 52242-5000, USA; b University of Kentucky, Department of Chemistry, Lexington, KY 40506-0055, USA

## Abstract

In the crystal structure of the title compound, C_6_H_2_Cl_2_INO_2_, there are weak C—H⋯Cl inter­actions and I⋯O [3.387 (4) Å] close contacts. These inter­actions form sheets in the *ac* plane, with the closest contact between adjacent planes occurring between inversion-related nitro O atoms [3.025 (8) Å]. The molecule possesses mirror symmetry, with the halogen, N and C atoms all lying in the mirror plane. Hence, the dihedral angle between the benzene ring and the nitro group is 90°.

## Related literature   

For crystal structures of similar substituted nitro­benzenes, see: Li *et al.* (2012 ▶); Tahir *et al.* (2009 ▶). For information about polychlorinated bi­phenyls (PCBs) and their synthesis, see: Joshi *et al.* (2011 ▶); Lehmler *et al.* (2010 ▶); Lehmler & Robertson (2001 ▶). For the synthesis of the title compound, see: Sohn *et al.* (2003 ▶).
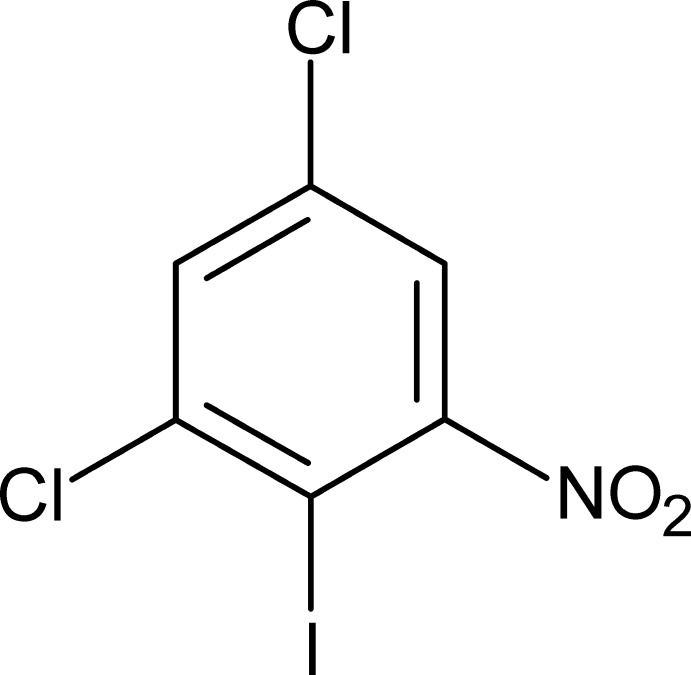


## Experimental   

### 

#### Crystal data  

C_6_H_2_Cl_2_INO_2_
*M* *_r_* = 317.89
Orthorhombic, 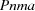

*a* = 8.7760 (5) Å
*b* = 6.8989 (4) Å
*c* = 14.3518 (8) Å
*V* = 868.93 (9) Å^3^
*Z* = 4
Cu *K*α radiation
μ = 34.30 mm^−1^
*T* = 90 K
0.13 × 0.10 × 0.04 mm

#### Data collection  

Bruker X8 Proteum diffractometer
Absorption correction: multi-scan (*SADABS*; Sheldrick, 2008*b* ▶) *T*_min_ = 0.052, *T*_max_ = 0.216
9625 measured reflections
862 independent reflections
827 reflections with *I* > 2σ(*I*)
*R*_int_ = 0.082

#### Refinement  

*R*[*F* ^2^ > 2σ(*F* ^2^)] = 0.037
*wR*(*F* ^2^) = 0.098
*S* = 1.12
862 reflections
71 parameters
H-atom parameters constrained
Δρ_max_ = 0.67 e Å^−3^
Δρ_min_ = −0.68 e Å^−3^

### 

Data collection: *APEX2* (Bruker, 2006) ▶; cell refinement: *SAINT* (Bruker, 2006) ▶; data reduction: *SAINT*; program(s) used to solve structure: *SHELXS97* (Sheldrick, 2008*a* ▶); program(s) used to refine structure: *SHELXL2013* (Sheldrick, 2008*a* ▶); molecular graphics: *XP in *SHELXTL** (Sheldrick, 2008*a* ▶); software used to prepare material for publication: *SHELXTL* and *CIFFIX* (Parkin, 2013 ▶).

## Supplementary Material

Crystal structure: contains datablock(s) I. DOI: 10.1107/S1600536814008733/lh5698sup1.cif

Structure factors: contains datablock(s) I. DOI: 10.1107/S1600536814008733/lh5698Isup2.hkl

Supporting information file. DOI: 10.1107/S1600536814008733/lh5698Isup3.cml

CCDC reference: 997807

Additional supporting information:  crystallographic information; 3D view; checkCIF report

## Figures and Tables

**Table 1:** Hydrogen-bond geometry (Å, °)

*D*—H⋯*A*	*D*—H	H⋯*A*	*D*⋯*A*	*D*—H⋯*A*
C3—H3⋯Cl2^i^	0.95	2.77	3.718 (7)	179

Symmetry code: (i) 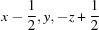.

**Fig. 1.**
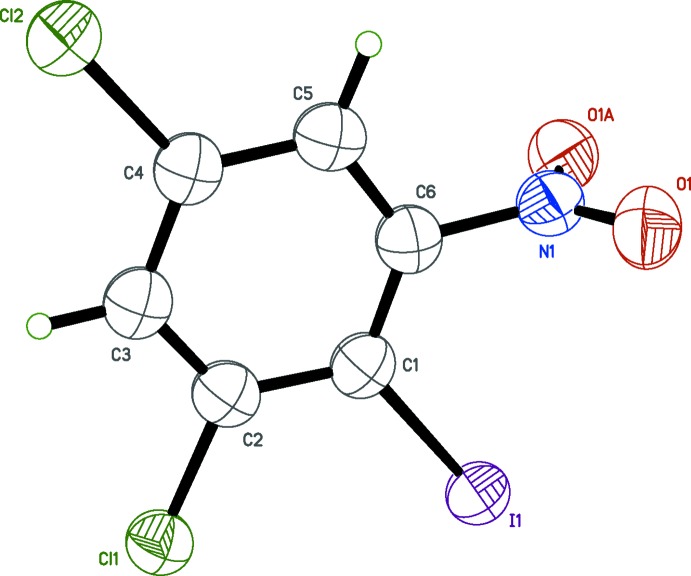
The molecular structure of the title compound, showing displacement ellipsoids drawn at the 50% probability level. Symmetry code: (A) x, -y+1/2, z.

## supplementary crystallographic information

### 1. Comment

The title compound was synthesized as a precursor for the preparation of chiral polychlorinated biphenyl (PCB) derivatives (Lehmler *et al.*, 2010) using the Suzuki-coupling reaction (Joshi *et al.*, 2011; Lehmler & Robertson, 2001). There are C3—H3···Cl2 (*x* - 0.5, *y*, 0.5 - *z*) interactions [C3···Cl2 = 3.718 (7) Å] that link the molecules into flat ribbons along the *a* axis. Between adjacent ribbons there are close contacts between iodine atoms and the nitro group O atoms, with I···O distances of 3.387 (4) Å. Each iodine atom is the same distance from both oxygen atoms because they are equivalent by virtue of the mirror plane. The linking of adjacent ribbons in the crystal structure give sheets in the *ac* plane (since the mirror plane is perpendicular to b). The closest contact between adjacent planes occurs between inversion (1 - *x*, 1 - *y*, 1 - *z*) related nitro O atoms [3.025 (8) Å]. The distance between layers is simply half the *b* axis length. Viewed along the *b* axis, molecules appear to stack in an alternating fashion about a 2_1_ screw (-*x*, 0.5 + *y*,-*z*), which places Cl1 of one molecule directly over the benzene ring of its screw-related counterpart.

As a result of the symmetrical interaction between the iodines and both nitro group O atoms, the molecular structure of the title compound displayed a 90° dihedral angle between the plane of the nitro group and the plane of the benzene ring (which lies on the mirror plane). Only a few solid state structures of structurally related molecules with a 1-iodo-2-nitrobenzene moiety have been reported previously. The molecular structures of 4-chloro-1-iodo-2-nitrobenzene, a structurally related halogenated nitrobenzene with one iodo substituent *ortho* to the nitro group, display smaller dihedral angles between benzene ring and nitro group [51.0 (3)° and 29.0 (2)°] in the solid state (Tahir *et al.*, 2009). In contrast, 2,4-diiodo-3-nitroanisole, a nitrobenzene with two iodo substituents *ortho* to the nitro group, displayed dihedral angle of 88.0 (3)° (Li *et al.*, 2012), probably due to the steric demand of the two *ortho* iodo substituents. These differences demonstrate that packing effects can make significant contributions to the molecular structure (*i.e.* the dihedral angle between benzene ring and nitro group) in the solid state.

### 2. Experimental

The title compound was synthesized from 2,4-dichloro-6-nitroaniline by sequential diazotization and iodonization with NaNO_2_–HCl–KI system (Sohn *et al.*, 2003). Crystals of the title compound suitable for crystal structure analysis were obtained by slow evaporation of a solution of the title compound in hexane-ethyl acetate (10:1).

### 3. Refinement

H atoms were found in difference Fourier maps, but subsequently included in the refinement using riding models, with constrained distances set to 0.95Å (C_sp2_H). *U*_iso_(H) values were set to 1.2*U*_eq_ of the attached atom.

### Crystal data

**Table:**

C_6_H_2_Cl_2_INO_2_	*D*_x_ = 2.430 Mg m^−^^3^
*M**_r_* = 317.89	Cu *K*α radiation, λ = 1.54178 Å
Orthorhombic, *P**n**m**a*	Cell parameters from 6956 reflections
*a* = 8.7760 (5) Å	θ = 5.9–67.7°
*b* = 6.8989 (4) Å	µ = 34.30 mm^−^^1^
*c* = 14.3518 (8) Å	*T* = 90 K
*V* = 868.93 (9) Å^3^	Rounded block, pale yellow
*Z* = 4	0.13 × 0.10 × 0.04 mm
*F*(000) = 592	

### Data collection

**Table:**

Bruker X8 Proteum diffractometer	862 independent reflections
Radiation source: fine-focus rotating anode	827 reflections with *I* > 2σ(*I*)
Detector resolution: 5.6 pixels mm^-1^	*R*_int_ = 0.082
φ and ω scans	θ_max_ = 68.0°, θ_min_ = 5.9°
Absorption correction: multi-scan (*SADABS*; Sheldrick, 2008*b*)	*h* = −10→7
*T*_min_ = 0.052, *T*_max_ = 0.216	*k* = −8→8
9625 measured reflections	*l* = −13→17

### Refinement

**Table:**

Refinement on *F*^2^	Secondary atom site location: difference Fourier map
Least-squares matrix: full	Hydrogen site location: difference Fourier map
*R*[*F*^2^ > 2σ(*F*^2^)] = 0.037	H-atom parameters constrained
*wR*(*F*^2^) = 0.098	*w* = 1/[σ^2^(*F*_o_^2^) + (0.0668*P*)^2^ + 0.2013*P*] where *P* = (*F*_o_^2^ + 2*F*_c_^2^)/3
*S* = 1.12	(Δ/σ)_max_ = 0.001
862 reflections	Δρ_max_ = 0.67 e Å^−^^3^
71 parameters	Δρ_min_ = −0.68 e Å^−^^3^
0 restraints	Extinction correction: *SHELXL2013* (Sheldrick, 2008a), Fc^*^=kFc[1+0.001xFc^2^λ^3^/sin(2θ)]^-1/4^
Primary atom site location: structure-invariant direct methods	Extinction coefficient: 0.0021 (4)

### Special details

**Table:**

Experimental. Diffraction data were collected with the crystal at 90 K, which is standard practice in this laboratory for the majority of flash-cooled crystals.A correction for radiation damage was included in the *SADABS* (Sheldrick, 2008*b*) run. This seems to have resulted in all the atomic displacement parameter ellipsoids looking more spherical than usual.
Geometry. All e.s.d.'s (except the e.s.d. in the dihedral angle between two l.s. planes) are estimated using the full covariance matrix. The cell e.s.d.'s are taken into account individually in the estimation of e.s.d.'s in distances, angles and torsion angles; correlations between e.s.d.'s in cell parameters are only used when they are defined by crystal symmetry. An approximate (isotropic) treatment of cell e.s.d.'s is used for estimating e.s.d.'s involving l.s. planes.

### Fractional atomic coordinates and isotropic or equivalent isotropic displacement parameters (Å^2^)

**Table:**

	*x*	*y*	*z*	*U*_iso_*/*U*_eq_	
I1	0.10397 (4)	0.2500	0.70325 (2)	0.0776 (3)	
Cl1	−0.18714 (15)	0.2500	0.54761 (10)	0.0783 (4)	
Cl2	0.16156 (18)	0.2500	0.24439 (10)	0.0807 (4)	
N1	0.4037 (5)	0.2500	0.5700 (4)	0.0797 (14)	
O1	0.4576 (4)	0.4066 (6)	0.5918 (2)	0.0946 (9)	
C1	0.1230 (7)	0.2500	0.5590 (5)	0.0739 (13)	
C2	−0.0053 (7)	0.2500	0.5003 (5)	0.0753 (13)	
C3	0.0065 (7)	0.2500	0.4060 (4)	0.0754 (13)	
H3	−0.0828	0.2500	0.3686	0.090*	
C4	0.1510 (8)	0.2500	0.3637 (5)	0.0740 (13)	
C5	0.2811 (7)	0.2500	0.4179 (5)	0.0765 (13)	
H5	0.3796	0.2500	0.3904	0.092*	
C6	0.2624 (7)	0.2500	0.5133 (4)	0.0757 (13)	

### Atomic displacement parameters (Å^2^)

**Table:**

	*U*^11^	*U*^22^	*U*^33^	*U*^12^	*U*^13^	*U*^23^
I1	0.0812 (4)	0.0798 (4)	0.0718 (4)	0.000	0.00182 (13)	0.000
Cl1	0.0753 (8)	0.0786 (8)	0.0810 (8)	0.000	0.0026 (6)	0.000
Cl2	0.0828 (9)	0.0878 (9)	0.0715 (8)	0.000	0.0000 (6)	0.000
N1	0.079 (3)	0.091 (4)	0.070 (3)	0.000	0.003 (2)	0.000
O1	0.0929 (19)	0.097 (2)	0.0936 (18)	−0.0143 (17)	−0.0113 (16)	−0.0056 (17)
C1	0.081 (3)	0.071 (3)	0.069 (3)	0.000	−0.001 (2)	0.000
C2	0.074 (3)	0.067 (3)	0.084 (3)	0.000	0.002 (3)	0.000
C3	0.084 (3)	0.065 (3)	0.077 (3)	0.000	−0.004 (3)	0.000
C4	0.079 (3)	0.069 (3)	0.074 (3)	0.000	−0.004 (3)	0.000
C5	0.076 (3)	0.073 (3)	0.081 (3)	0.000	0.005 (3)	0.000
C6	0.076 (3)	0.073 (3)	0.078 (3)	0.000	−0.003 (3)	0.000

### Geometric parameters (Å, º)

**Table:**

I1—C1	2.077 (7)	C1—C2	1.406 (9)
Cl1—C2	1.734 (7)	C2—C3	1.358 (9)
Cl2—C4	1.715 (7)	C3—C4	1.406 (10)
N1—O1	1.220 (4)	C3—H3	0.9500
N1—O1^i^	1.220 (4)	C4—C5	1.381 (9)
N1—C6	1.484 (8)	C5—C6	1.379 (9)
C1—C6	1.388 (9)	C5—H5	0.9500
			
O1—N1—O1^i^	124.6 (6)	C4—C3—H3	120.0
O1—N1—C6	117.7 (3)	C5—C4—C3	120.2 (6)
O1^i^—N1—C6	117.7 (3)	C5—C4—Cl2	121.1 (5)
C6—C1—C2	114.9 (6)	C3—C4—Cl2	118.7 (5)
C6—C1—I1	122.9 (5)	C6—C5—C4	117.4 (6)
C2—C1—I1	122.2 (5)	C6—C5—H5	121.3
C3—C2—C1	122.4 (6)	C4—C5—H5	121.3
C3—C2—Cl1	117.4 (5)	C5—C6—C1	125.1 (6)
C1—C2—Cl1	120.2 (5)	C5—C6—N1	116.5 (5)
C2—C3—C4	119.9 (6)	C1—C6—N1	118.4 (5)
C2—C3—H3	120.0		
			
C6—C1—C2—C3	0.000 (2)	C4—C5—C6—C1	0.000 (2)
I1—C1—C2—C3	180.000 (1)	C4—C5—C6—N1	180.000 (1)
C6—C1—C2—Cl1	180.000 (1)	C2—C1—C6—C5	0.000 (2)
I1—C1—C2—Cl1	0.000 (1)	I1—C1—C6—C5	180.000 (1)
C1—C2—C3—C4	0.000 (2)	C2—C1—C6—N1	180.000 (1)
Cl1—C2—C3—C4	180.000 (1)	I1—C1—C6—N1	0.000 (2)
C2—C3—C4—C5	0.000 (2)	O1—N1—C6—C5	90.0 (5)
C2—C3—C4—Cl2	180.000 (1)	O1^i^—N1—C6—C5	−90.0 (5)
C3—C4—C5—C6	0.000 (1)	O1—N1—C6—C1	−90.0 (5)
Cl2—C4—C5—C6	180.000 (1)	O1^i^—N1—C6—C1	90.0 (5)

Symmetry code: (i) *x*, −*y*+1/2, *z*.

### Hydrogen-bond geometry (Å, º)

**Table:**

*D*—H···*A*	*D*—H	H···*A*	*D*···*A*	*D*—H···*A*
C3—H3···Cl2^ii^	0.95	2.77	3.718 (7)	179

Symmetry code: (ii) *x*−1/2, *y*, −*z*+1/2.

## References

[bb1] Bruker (2006). *APEX2* Bruker AXS Inc., Madison, Wisconsin, USA.

[bb2] Joshi, S. N., Vyas, S. M., Duffel, M. W., Parkin, S. & Lehmler, H.-J. (2011). *Synthesis*, pp. 1045–1054. 10.1055/s-0030-1258454 PMC3080045 21516177

[bb3] Lehmler, H.-J., Harrad, S. J., Huhnerfuss, H., Kania-Korwel, I., Lee, C. M., Lu, Z. & Wong, C. S. (2010). *Environ. Sci. Technol.* **44**, 2757–2766. 10.1021/es902208u PMC2855137 20384371

[bb4] Lehmler, H.-J. & Robertson, L. W. (2001). *Chemosphere*, **45**, 137–143. 10.1016/s0045-6535(00)00546-4 11572605

[bb5] Li, X., Cao, L., Ruan, C., Ji, B. & Zhou, L. (2012). *Acta Cryst.* E**68**, o1500. 10.1107/S160053681200952X PMC3344609 22590371

[bb6] Parkin, S. (2013). *CIFFIX* http://xray.uky.edu/people/parkin/programs/ciffix.

[bb7] Sheldrick, G. M. (2008*a*). *Acta Cryst.* **A**64, 112–122.

[bb8] Sheldrick, G. M. (2008*b*). *SADABS* University of Göttingen, Germany.

[bb9] Sohn, J., Kiburz, B., Li, Z., Deng, L., Safi, A., Pirrung, M. C. & Rudolph, J. (2003). *J. Med. Chem.* **46**, 2580–2588. 10.1021/jm0300835 12801222

[bb10] Tahir, M. N., Arshad, M. N., Khan, I. U. & Shafiq, M. (2009). *Acta Cryst.* E**65**, o535. 10.1107/S1600536809004930 PMC2968683 21582195

